# Interference by new-generation mobile phones on critical care medical equipment

**DOI:** 10.1186/cc6115

**Published:** 2007-09-06

**Authors:** Erik Jan van Lieshout, Sabine N van der Veer, Reinout Hensbroek, Johanna C Korevaar, Margreeth B Vroom, Marcus J Schultz

**Affiliations:** 1 Department of Intensive Care Medicine, Academic Medical Centre, University of Amsterdam, Meibergdreef 9, 1105 AZ Amsterdam, The Netherlands; 2 Mobile Intensive Care Unit, Academic Medical Centre, University of Amsterdam, Meibergdreef 9, 1105 AZ Amsterdam, The Netherlands; 3 Department of Medical Engineering, Academic Medical Centre, University of Amsterdam, Meibergdreef 9, 1105 AZ Amsterdam, The Netherlands; 4 Department of Prevention and Health, Netherlands Organisation for Applied Scientific Research, Zernikedreef 9, 2333 CK Leiden, The Netherlands; 5 Department of Clinical Epidemiology, Biostatistics and Bioinformatics, Academic Medical Centre, University of Amsterdam, Meibergdreef 9, 1105 AZ Amsterdam, The Netherlands; 6 Laboratory of Experimental Intensive Care and Anaesthesiology, Academic Medical Centre, University of Amsterdam, Meibergdreef 9, 1105 AZ Amsterdam, The Netherlands

## Abstract

**Introduction:**

The aim of the study was to assess and classify incidents of electromagnetic interference (EMI) by second-generation and third-generation mobile phones on critical care medical equipment.

**Methods:**

EMI was assessed with two General Packet Radio Service (GPRS) signals (900 MHz, 2 W, two different time-slot occupations) and one Universal Mobile Telecommunications System (UMTS) signal (1,947.2 MHz, 0.2 W), corresponding to maximal transmit performance of mobile phones in daily practice, generated under controlled conditions in the proximity of 61 medical devices. Incidents of EMI were classified in accordance with an adjusted critical care event scale.

**Results:**

A total of 61 medical devices in 17 categories (27 different manufacturers) were tested and demonstrated 48 incidents in 26 devices (43%); 16 (33%) were classified as hazardous, 20 (42%) as significant and 12 (25%) as light. The GPRS-1 signal induced the most EMI incidents (41%), the GRPS-2 signal induced fewer (25%) and the UMTS signal induced the least (13%; *P *< 0.001). The median distance between antenna and medical device for EMI incidents was 3 cm (range 0.1 to 500 cm). One hazardous incident occurred beyond 100 cm (in a ventilator with GRPS-1 signal at 300 cm).

**Conclusion:**

Critical care equipment is vulnerable to EMI by new-generation wireless telecommunication technologies with median distances of about 3 cm. The policy to keep mobile phones '1 meter' from the critical care bedside in combination with easily accessed areas of unrestricted use still seems warranted.

## Introduction

Electromagnetic interference (EMI) with medical equipment by second-generation mobile phones has been reported extensively and seems clinically relevant to about 10% of medical devices [1-7]. The growth in use and the decrease in size of mobile phones intensifies the discussion on present hospital restrictions on the use of mobile phones in patient areas, which is violated by healthcare workers themselves to improve patient care by better communication [8]. Critical incidents caused by mobile phones are probably rare but are potentially lethal and are most probably not recognized as such [9,10].

First-generation mobile phones are mainly used for voice, whereas new generations of telecommunication systems enable us to have wireless internet access to send and receive data even at the patient's bedside [11]. Data transmission may be of more concern in the context of EMI. However, these new systems entered the market with limited proof of their safety in the critical care environment [12]. Unfortunately, studies on EMI-induced incidents are characterized by a technical description of incidents only, whereas classification of their clinical relevance is needed to update evidence-based policies on the use of modern mobile phones [3,13].

The aim of the present study was to assess and classify incidents of EMI by second-generation and third-generation telecommunication signals on 61 critical care devices.

## Methods

### Medical equipment

In all, 61 different medical devices (27 different manufacturers) in 17 categories were allocated for EMI tests (Table 1). The details of the devices are summarized in Additional file 1. All devices were tested in accordance with an international test protocol during full operation and in different modes; a simulator (namely an electrocardiogram simulator, an artificial lung and a syringe filled with saline) was connected if relevant [14]. The tests were performed on devices in use for patient care by two different hospitals (Academic Medical Center, Amsterdam, The Netherlands, and Kennemer Gasthuis, Haarlem, The Netherlands) to maximize the number of devices; similar test conditions were used in each location.

**Table 1:** Categories of medical devices, interference distances and type of incidents per signal

Type of device or incident	Number of devices		Distance^a ^(cm)	Type of incident per signal^b^		
			
	Tested	Influenced		GPRS-1	GPRS-2	UMTS
Intensive care unit ventilator	9	7	1.5 [0.1–300]	6H, 1L	2H, 1S, 1L	1H, 2S, 1L
Critical care monitor	13	7	3 [0.1–500]	4S, 3L	2S, 4L	
Syringe pump	7	3	5 [0.1–50]	2H, 1S	S	S
Volumetric infusion pump	4	1	30	S	S	S
Intra-aortic balloon pump	2	1	0.1	L		
Haemofiltration/dialysis	5	1	15	H		
External pacemaker	4	1	3	H		
Defibrillator	3	1	0.1			L
12-lead EKG	1	1	150	S	S	S
Fluid warmer	2	1	6	S	S	
Enteral feeding pump	2	1	30	H	H	
Air humidifier	1	1	5	H		
EKG telemetry	1	0				
Forced-air warming unit	3	0				
Mobile suction unit	1	0				
Critical care bed	2	0				
Continuous-airflow mattress	1	0				
Type of incident^b^						
Hazardous			3.5 [0.1–300]			
Significant			25 [0.1–500]			
Light			0.1 [0.1–3]			
Total	61	26 (43%)	3 [0.1–500]	25 (41%)	15 (25%)	8 (13%)

GPRS, General Packet Radio Service; UMTS, Universal Mobile Telecommunications System; EKG, electrocardiogram. ^a^Results are shown as median [range]. ^b^Hazardous (H) is defined as a direct physical influence on patient by unintended change in equipment function; significant (S) is defined as an influence on monitoring with a significant level of attention needed, causing substantial distraction from patient care; light (L) is defined as an influence on monitoring without a significant level of attention needed.

### Signals

The General Packet Radio Service (GPRS) signals had time-slot durations of 1,113 μs and a repetition frequency of 217 Hz (GRPS-1) or 556.5 μs at 27.1 Hz (GPRS-2), both with a 0.2 MHz channel bandwidth and a carrier frequency of 900 MHz. This GPRS technology, based on time-division multiple-access technology and available for data transfer in Europe, the United States, Australia and parts of Asia, was chosen for its forthcoming use for data transmission [11]. GPRS is considered a 2.5-generation wireless telephony system.

The Universal Mobile Telecommunications System (UMTS) signal had a bandwidth of 5 MHz and a carrier frequency of 1,947.2 MHz. This wideband code-division multiple-access frequency-division duplex technology is considered a third-generation wireless telephony system. A signal generator (HP/Agilent E4433B/ESG-D Digital RF 250 kHz to 4 GHz), provided with a Global System for Mobile Communications (GSM)/W-CDMA module, was used in combination with external control equipment (a laptop and an additional pulse generator) for timing purposes. The signals were amplified and their power level was controlled at 2 W for GRPS in active time slots and at 0.2 W for UMTS. These power levels correspond to maximal transmit performance of mobile phones in daily practice and were chosen to mimic a worst-case but realistic scenario to maximize the chance of detecting EMI-related incidents.

The signals were radiated towards the medical apparatus through an electrically balanced handheld antenna without reflecting obstacles nearby. Special attention was paid to poorly shielded locations in device housings (such as connectors, sensors, and seams in the housing). The initial distance between antenna and device was 500 cm from the device housing and was decreased to 0 cm or until any incident occurred [14]. In the event of any interference the test was repeated three times to assess reproducibility.

### Classification of incidents

Incidents observed during the normal operation of each device were documented in detail. Two board-certified and experienced intensivists classified by consensus of opinions the severity of the observed incidents in accordance with an adjusted scale of critical care adverse events [15]. The scale ranges from light (influence on monitoring without a significant level of attention needed, for example a disturbed display) through significant (influence on monitoring with a significant level of attention needed, causing substantial distraction from patient care, for example an incorrect alarm or inaccurate monitoring of blood pressure) to hazardous (direct physical influence on the patient by an unintended change in equipment function, for example total stopping of ventilator or syringe pump).

### Statistical analysis

Median, maximum and minimum are given if no normal distribution was established. Distances are expressed in centimetres. The distance between the antenna and device was set at 0.1 cm if an incident occurred when the antenna was held against the housing of the device. Percentages of critical care devices disturbed by second-generation and third-generation telecommunication signals (GPRS-1, GPRS-2 and UMTS) were compared by using Cochran's *Q *test. The difference between median distances between antenna and device at which incidents occurred were analysed with the Friedman test. A linear-by-linear χ^2 ^test was performed to test for a trend in the frequency of incidents in relation to the year of purchase of the device.

## Results

EMI by GPRS or UMTS signals on critical care medical equipment was demonstrated in 26 of the 61 device tests (43%) (Table 1). A total of 48 incidents were identified and classified as 16 (33%) hazardous, 20 (42%) significant and 12 (25%) light.

The GPRS-1 signal induced the highest number of incidents of EMI: 41% (25 of 61), followed by GRPS-2 (25%; 15 of 61) and UMTS (13%; 8 of 61; *P *< 0.001). The same was true of the hazardous incidents: GPRS-1 20% (12 of 61), GPRS-2 5% (3 of 61) and UMTS 2% (1 of 61; *P *< 0.001). The medical devices and descriptions of all incidents are listed in Additional file 1.

Hazardous incidents occurred in devices for therapy only due to the definitions of the adjusted critical adverse events scale. In mechanical ventilators, nine hazardous incidents (in seven ventilators out of nine tested; median distance 3 cm, range 0.1 to 300) varied from 'total switch-off and restart' to changes in set ventilation rate. In syringe pumps, two hazardous incidents (in two pumps out of seven tested; distances 0.1 and 2 cm) demonstrated a complete stop without an acoustic alarm or with an incorrect alarm. One hazardous incident in a renal replacement device (out of five machines tested; distance 15 cm) showed a stop after an incorrect air detector alarm. One external pacemaker (out of three tested; distance 3 cm) demonstrated a hazardous incident, with incorrect inhibition of the pacemaker.

**Figure 1:**
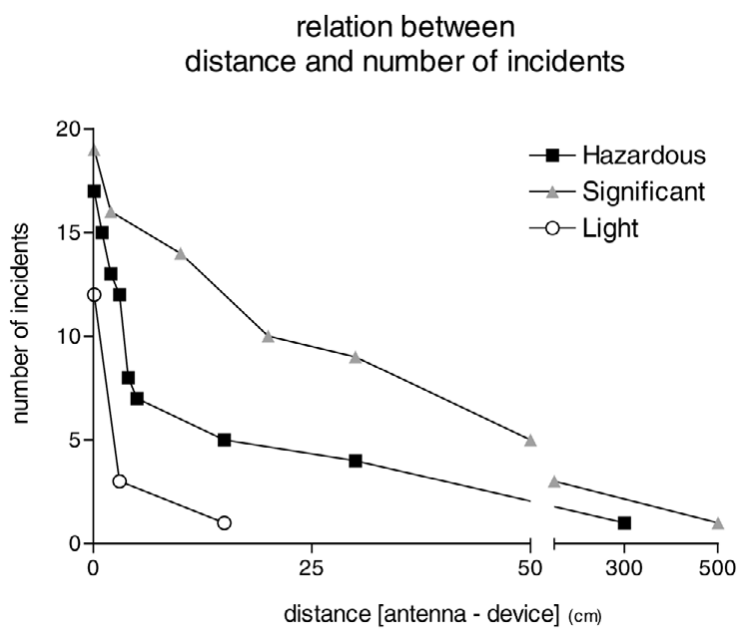
Relation between distance and number of incidents.

The median distance between antenna and device at which all type of incident occurred was 3 cm, range (0.1 to 500 cm). The relation between distance and number of hazardous, light and significant incidents is depicted in Figure 1.

Incidents occurred at greater distance with the GPRS-1 signal (median 5 cm) than with the GPRS-2 (median 3 cm) or UMTS (median 1 cm) signal, although the differences were not statistically significant (*P *= 0.12).

Hazardous incidents occurred at a median distance of 3.5 cm (range 0.1 to 300 cm). Beyond 100 cm one hazardous incident at 300 cm in a ventilator with the GRPS-1 signal and two significant incidents occurred at 150 cm in a 12-lead electrocardiogram device with GPRS 1, GPRS-2 and UMTS signals (see Additional file 1).

No relation could be demonstrated between the year of purchase of medical devices and the number of incidents (*P *= 0.67).

## Discussion

The present study demonstrates two new findings in the field of interference by mobile phones on medical equipment.

First, the 2.5-generation mobile communication network GPRS is able to induce a higher rate of EMI incidents than is known for the first-generation network GSM at comparable distances [1,3,7]. Second, the median distance at which EMI incidents caused by new-generation cellular phones take place (3 cm) falls within the '1 meter rule' proposed as a safe distance in patient areas, although the range demonstrated in this study is considerable (0.1 to 500 cm) [1,5,11,16].

Studies on EMI by first-generation mobile phones have been based on the GSM network used in Europe, the United States, Australia and part of Asia, or on code-division multiple access (CDMA), which is used mostly in the United States [2,3]. Meanwhile GPRS and UMTS networks are used for their advanced properties to transmit video and data wirelessly at a higher speed as well as regular voice telephony [12].

Our finding of EMI induced by UMTS with hazardous incidents contrasts with what was demonstrated recently in the only study so far on UMTS by Wallin and colleagues [12]. No critical UMTS incidents with 76 medical devices were reported besides interference noise on loudspeakers of two ultrasonic Doppler devices. Their only critical incident with GPRS was the total stopping of one infusion pump (out of 12 tested) at a distance of 50 cm. Neither GPRS nor UMTS demonstrated any interference on four intensive care ventilators tested. Three of those ventilators were also tested in our study, and in contrast with those studied by Wallin and colleagues they showed significant and hazardous GRPS incidents and one light UMTS incident. There are two possible explanations for these differences. First, Wallin and colleagues used a different GPRS signal with a frequency of 1,800 MHz and an output power of 1 W, as opposed to 900 MHz and 2 W used in the present study. The lower carrier-wave frequency of the GPRS signal and the corresponding 2 W in our study was chosen for its availability in many continents. GPRS is used worldwide on different frequency bands (900 and 1,800 MHz) in different continents and therefore many 'tri-band or quad-band' mobile phones are sold for their worldwide operation [3,13]. Second, the studies differed in their selection from medical equipment available worldwide. Our results apply to the tested devices only as specified, including the year of purchase, and consequently are a limitation of the present study.

Another limitation of this study is the test conditions. The only method for obtaining reproducible results in testing EMI by mobile phones is a standard signal generator to control output power as used in the study by Wallin and colleagues and in our own [3,12]. The use of commercially available mobile phones in ringing mode will generate irreproducible results at different locations because mobile phones (GSM, GPRS and UMTS) regulate their output power depending on the nearest cell base station for the telecom provider [4,17]. If such a station is nearby, a mobile phone constantly minimizes its required output power, in GPRS to as low as 5 to 10% (50 to 100 mW), to increase its battery lifespan. In our study the output power was controlled and set at the maximum level to mimic a worst-case but realistic scenario. In healthcare facilities the coverage of telecommunication networks could be poor because of its structures and could consequently induce mobile phones to transmit at maximum power, which increases the risk of EMI [1,12]. Therefore, as a result of our worst-case scenario it is not to be expected that in daily practice critical EMI incidents with GPRS or UMTS would be more frequent than reported in our study.

Health care applications of new wireless telecommunication technologies are reaching the bedside (namely intelligent pager systems with smart phones, personal digital assistants with internet access, and telemonitoring interhospital intensive care transport) with potential clinical benefits [2,8]. However, critical care equipment, with closed loop systems to eliminate human resources and errors, demands permanent technology assessment to ensure its continued performance including electromagnetic compatibility with other devices [2].

The international standard on electromagnetic compatibility by the International Electrotechnical Commission in its present form is insufficient to safeguard medical equipment completely from EMI by GSM mobile phones, and our results show that the same holds true for GPRS and UMTS signals [11,18]. The present industrial standard lacks stipulations for eliminating EMI in medical equipment. Manufacturers are allowed to comply with the standard by reporting only the distance at which EMI occurs. Reasons why even new medical devices still demonstrate EMI caused by mobile phones would be speculative; examples are complex medical industrial design, rapidly changing telecommunications signals, and costs. This leads one to suspect that the undesirable situation of EMI in the critical care environment will not be eradicated soon.

This study adds to the objective evidence that restrictive use in the critical care environment is sensible without overstressing negligible risks [11,19].

## Conclusion

The '1 meter rule', specifying the minimum distance to keep a mobile phone from medical equipment or the bedside as proposed in the past, seems safe, although the rule does not exclude EMI by new-generation mobile phones entirely. Restrictive policies should be facilitated by offering numerous areas that are easily accessed throughout the healthcare facility where the use of mobile phones is clearly permitted.

## Key messages

• Incidents of EMI caused by second-generation and third-generation mobile phones occurred in 43% of 61 critical care medical devices, of which 33% were classified as hazardous.

• The hazardous incidents varied from a total switch-off and restart of a mechanical ventilator, through complete stops without alarms in syringe pumps, to incorrect pulsing by an external pacemaker.

• The median distance of all incidents was 3 cm, with a considerable range up to 500 cm.

• The policy to keep mobile phones '1 meter' from the critical care bedside in combination with easily accessed areas of unrestricted use still seems warranted.

## Abbreviations

CDMA = code-division multiple access; EMI = electromagnetic interference; GPRS = General Packet Radio Service; GSM = Global System for Mobile Communications; UMTS = Universal Mobile Telecommunications System.

## Competing interests

The authors declare that they have no competing interests.

## Authors' contributions

EJvL designed the study, performed the measurements, assisted in the statistical analyses and drafted the manuscript. SNvdV designed the study, helped in performing the measurements and interpreting the results and participated in drafting the manuscript. RH designed the study, performed the measurements and participated in drafting the manuscript. JCK performed the statistical analysis and participated in drafting the manuscript. MBV and MJS participated in the study design, in interpreting the results and in drafting the manuscript. All authors read and approved the final manuscript.

## Supplementary Material

Additional file 1 An Excel file containing a list of medical devices and descriptions of all incidents. Click here for file
